# Real-world data on efficacy and safety of azacitidine therapy in chronic myelomonocytic leukemia in China: results from a multicenter, retrospective study

**DOI:** 10.1007/s10637-022-01283-x

**Published:** 2022-07-14

**Authors:** Yu Xu, Rong Guo, Miao Miao, Guangsen Zhang, Jianping Lan, Jie Jin

**Affiliations:** 1 Department of Hematology, First Affiliated Hospital，Zhejiang University School of Medicine, Hangzhou, 310003 China; 2 Department of Hematology, The First Affiliated Hospital of Zhengzhou University, Zhengzhou, 450052 China; 3 Department of Hematology, The First Affiliated Hospital of Soochow University, Suzhou, 215006 China; 4 Departement of Hematology, The Second Xiangya Hospital of Central South University, Changsha, 430011 China; 5 Department of Hematology, Zhejiang Provincial People’s Hospital, People’s Hospital of Hangzhou Medical College, Hangzhou, 310014 China

**Keywords:** Chronic myelomonocytic leukemia, Hypomethylating agents, Azacitidine, Efficacy, Tolerability

## Abstract

Chronic myelomonocytic leukemia (CMML) is a rare and aggressive myeloid malignancy with overlapped features of myelodysplastic syndromes/myeloproliferative neoplasms. Azacitidine (AZA), a hypomethylating agent, has been approved for the treatment of CMML in China, but real-world data are limited. Medical records of CMML patients who had received subcutaneously injected AZA were reviewed from January 2018 at five participating sites in China. Response was assessed according to the modified International Working Group (IWG 2006) criteria. Between January 2018 and November 2020, a total of 24 patients with CMML were included with a median age of 63 years. Patients received a median of 3 cycles of AZA treatment (range, 1–8). Overall response rate (ORR) was 37.5% (9 of 24); CR rate, PR rate, and mCR/HI rate were 8.3% (n = 2), 8.3% (n = 2), and 20.8% (n = 5), respectively. At a median duration of follow-up of 14.0 months (range 0.0–22.0 months), the median overall survival (OS) was 23.0 months. Univariate analysis revealed that ≥ 3 cycles of treatment was significantly associated with a higher 1-year OS rate compared with < 3 cycles of AZA treatment. Treatment was generally well-tolerated. The most common (> 10%) AEs were thrombocytopenia (n = 7, 29.2%), pneumonitis (n = 4, 16.7%) and fever (n = 3, 12.5%). This study provides valuable real-life data in China on the treatment schedules, efficacy and safety of AZA in the treatment of CMML.

## Introduction

Chronic myelomonocytic leukemia (CMML) is a clonal hematopoietic stem cell disorder with overlapping features of myelodysplastic syndromes (MDS) and myeloproliferative neoplasms (MPN) [1]. CMML is defined as having a blood monocyte count of at least 1 × 10^9^/L, which accounts for at least 10% of the white blood cell differential count (WBC), together with the presence of myelodysplastic and myeloproliferative alterations in the bone marrow [1, 2]. CMML has been originally considered a myelodysplastic syndrome in the 1994 French–American–British (FAB) classification, and subsequently classified into two subtypes on the basis of WBC count: myeloproliferative CMML (MP-CMML) with > 13 × 10^9^/L WBC count and myelodysplastic CMML (MD-CMML) with ≤ 13 × 10^9^/L WBC count [3–5]. Based on the blast cell portion in peripheral blood (PB) and bone marrow (BM), the 2016 WHO criteria classified CMML into CMML-0 (PB blasts < 2%, BM < 5% blasts), CMML-1 (PB blasts: 2–4%, BM blasts: 5–9%) and CMML-2 (PB blasts: 5–19%, BM blasts: 10–19%) [1].

CMML is a rare disease with an incidence of 0.3 per 100 000 in western counties [6–10]. It typically occurs in the elderly with a median age at diagnosis of 72–76 years and a male:female ratio of (1.5–3):1 [6, 7, 11–13].The prognosis of this aggressive myeloid malignancy is poor with a median survival of only 12 to 29 months and a 14–29% risk of acute leukemic transformation [14–19].

Allogeneic stem cell transplantation (allo-SCT) is the only potentially curative therapy, but only 10% are eligible considering the advanced age at diagnosis and comorbidities for patients with CMML [16]. Cytoreductive therapy and hypomethylating agents (HMA) remain the main therapeutic strategies for CMML.

Azacitidine (5-azacitidine, AZA) is an analogue of cytidine which inhibits DNA methyltransferases, resulting in the reactivation of epigenetically silenced suppressor genes [20, 21]. It has been approved by the Food and Drug Administration (FDA) for CMML treatment based on phase 3 randomized trials dedicated to MDS with a limited number of CMML patients [22, 23]. Till then, evidence of the effect of AZA in CMML is accumulated from local, small, non-randomized clinical trials and retrospective real-world experiences [24–29]. Since its approval by the China National Medical Products Administration (NMPA) in late 2017, real-world data on the efficacy of AZA in the treatment of CMML in China have been rather limited. A recent single-center retrospective study [30] highlighted the benefit of HMAs therapy (both monotherapy and combination therapy) in CMML patients in China with prolonged overall survival of 23.57 months compared with the chemotherapy (11.73 months) alone, but most patients received decitabine as HMA therapy with only 9 patients receiving AZA. Given the limited data on AZA treatment in China, we conducted this retrospective observational study to evaluate the efficacy and safety of AZA response to the treatment of CMML from multiple sites in China.

## Materials and methods

### Patients

This multicenter, retrospective study reviewed medical records of CMML patients who had received subcutaneously injected AZA from January 2018 at five participating sites in China. Eligible patients were ≥ 18 years of age, had CMML diagnosed by FAB classification or 2016 WHO classification, and had completed AZA treatment based on a prespecified protocol. Patients were excluded if they had other cancer diagnoses before or at the same time when they were diagnosed with CMML, or if they participated in interventional clinical trials of AZA or other drugs in CMML treatment. This study was conducted in accordance with the ethical standards of the institutional and/or national research committee and the guidelines established by the Helsinki Declaration.

We reviewed demographic and clinical data of patients including sex, age, ECOG performance status, date of initial diagnosis, past disease history, disease type by WHO or FAB classification, etc.

### Administration and response criteria

Treatment was at physicians’ discretion, with azacitidine prescribed according to local clinical practice. The dosage, frequency, and cycles of AZA administration were reviewed. Concomitant and other supportive therapy were also analyzed. Bone marrow and peripheral blood samples were collected at diagnosis and after completion of each treatment cycle or before the start of the next cycle, to evaluate morphology response and cytogenetic analysis. Cytogenetic analysis was performed by Polymerase Chain Reaction, and/or chromosomal g-banding, and/or fluorescent in situ hybridization in the Institute of Hematology at each site following standard protocols. Treatment response was assessed by physicians according to the modified International Working Group (IWG) criteria [31]. The overall response rate (ORR) was the sum of CR, PR, and mCR/HI.

### Follow-up

The last follow-up was performed on April 7, 2021. Survival was measured from the start of therapy to the day of death regardless of any cause or last contact, and follow-up for survival was carried out via telephone contact.

### Statistical analysis

Statistical analysis was performed using SAS software (version 9.4). Descriptive statistics were reported as median (range) for continuous variables, and frequencies and percentages for categorical variables. Overall survival (OS), progression-free survival (PFS), time-to-treatment response (TTR) and duration of response (DOR) were estimated using Kaplan–Meier analysis; the Brookmeyer and Crowley method was used to construct 95% CIs for the median of those time-to-event endpoints. Factors associated with OS were analyzed with univariate analysis due to the limited sample size. *P* value < 0.05 was considered statistically significant. No adjustment was used for multiple testing.

## Results

### Patient characteristics

Between January 2018 and November 2020, a total of 24 patients with CMML, 20 males (83.3%) and 4 females (16.7%), were included in this study with a median age of 63 years (range 22–93 years). The baseline characteristics are shown in Table 1. 21 of 24 (87.5%) patients were treated with AZA at first diagnosis, and the remaining 3 (12.5%) patients had relapsed/refractory CMML. Fourteen (58.3%) of 24 patients had MP-CMML, and the other 10 (41.7%) had MD-CMML according to FAB classification. According to 2016 WHO classification, 7 (29.2%) patients had CMML-0, 5 (20.8%) had CMML-1, and 12 (50.0%) had CMML-2. All patients underwent bone marrow aspiration, of which 7 (30.4%) were found to have dyshaematopoiesis. Twenty-three (95.8%) patients received cytogenetic evaluation, and frequently revealed a normal karyotype (15 patients, 62.5%). Aberrant karyotype was observed in 8 patients, accounting for 33.3% of the cohort, including 2 (8.3%) patients with complex karyotype and 2 (8.3%) with trisomy 8 abnormalities. 18 (75.0%) patients underwent molecular biological evaluation, and TET2, SRSF2 and ASXL1 mutation were detected in 5 (20.8%), 3 (12.5%) and 6 (25.0%) patients, respectively.

**Table 1:** Demographics and baseline characteristics of patients

**Characteristic**	**Patients (n = 24)**
**Median age, years (range)**	63 (22.0–93.0)
**Sex, n (%)**	
Male	20 (83.3)
Female	4 (16.7)
**FAB classification, n (%)**	
MP-CMML	14 (58.3)
MD-CMML	10 (41.7)
**2016 WHO classification, n (%)**	
CMML-0	7 (29.2)
CMML-1	5 (20.8)
CMML-2	12 (50.0)
**Baseline disease status, n (%)**	
Newly onset CMML	21 (87.5)
Relapse/refractory CMML	3 (12.5)
**Time from CMML diagnosis to azacitidine treatment, median, days (range)**	13.5 (1.0–1231.0)
**Blood counts, median (range)**	
WBC (× 10^9^/L)	8.8 (0.9–72.4)
Hemoglobin (g/L)	81.5 (2.0–140.0)
Platelets (× 10^9^/L)	54 (5.0–399.0)
Lymphocyte	1.5 (0.5–8.6)
Monocyte	2.8 (0.3–24.8)
**Morbid hematopoiesis, n (%)**	7 (30.4)
**Cytogenetics, n (%)**	
Normal karyotype	15 (62.5)
Monosomal karyotype	1 (4.2)
Complex karyotype	2 (8.3)
Trisomy 8	2 (8.3)
Chromosome 7 abnormalities (-7, del7q)	1 (4.2)
-Y or der(3q)	0 (0.0)
Other	3 (12.5)
**Mutated genes, n (%)**	
TET2	5 (20.8)
SRSF2	3 (12.5)
ASXL1	6 (25.0)
TP53	1 (4.2)
Other	16 (66.7)
**Azacitidine dose**	
75 mg/m^2^/d	9 (37.5)
100 mg/d fixed dose	15 (67.5)
**Concomitant medication**	
None	11 (45.8)
Chemotherapy	7 (29.2)
Lenalidomide	2 (8.3)
Venetoclax	2 (8.3)
Unknown	3 (12.5)

*CMML* chronic myelomonocytic leukemia, *MP-CMML* myeloproliferative CMML, *MD-CMML* myelodysplastic CMML, *WBC* white blood cell count

### Treatment administration

The median time from diagnosis to AZA treatment initiation was 13.5 (range, 1.0 -1231.0) days. A total of 9 (37.5%) patients received AZA at a dose of 75 mg/m^2^/day for 7 consecutive days; the other 15 (62.5%) patients received a fixed dose of 100 mg daily for a median of 7 consecutive days (range, 5–10 days) for each cycle (Table 2). The treatment was repeated every 28 days. The median number of cycles of AZA treatment was 3 (range, 1–8) cycles, and longer cycles of treatment were observed in patients receiving a fixed dose of AZA (median, 4 cycles) compared with body surface area-based administration (BSA, median, 3 cycles). Five (20.8%) patients completed 6 cycles of treatment, and all these patients were treated with a fixed daily dose of 100 mg AZA. Six (25.0%) patients received < 3 cycles of AZA treatment, including 3 patients who had stable disease after the first cycle of AZA, and 3 patients lost to follow-up. Nearly half (n = 11, 45.8%) of patients received AZA as monotherapy; the other half received AZA combination therapy, including 7 (29.2%) with AZA plus chemotherapy [hydroxycarbamide (HC), n = 4; HC and cytarabine, n = 1; venetoclax and cytarabine, n = 1; HC, G-CSF, homoharringtonine, and cytarabine, n = 1], 2 (8.3%) with AZA plus lenalidomide, 2 (8.3%) with AZA plus venetoclax [one was also received chemotherapy (cytarabine)].

**Table 2:** Treatment response after azacitidine treatment according to the modified International Working Group (IWG 2006) criteria

**Response**	**n (%)**	**(95% CI) (%)**
**CR**	2 (8.3)	(1.0, 27.0)
**PR**	2 (8.3)	(1.0, 27.0)
**SD**	5 (20.8)	(7.1, 42.2)
**PD**	5 (20.8)	(7.1, 42.2)
**mCR/HI**	5 (20.8)	(7.1, 42.2)
**Missing (unknown)**	5 (20.8)	-
**ORR (CR + PR + mCR/HI)**	9 (37.5)	(18.8, 59.4)

*CR* complete remission, *PR* partial remission, *SD* stable disease, *PD* progressive disease, *mCR/HI* marrow complete remission/hematologic improvement, *ORR* overall response rate

### Treatment outcome

Overall, 9 of 24 patients achieved response with an ORR of 37.5% (95%CI, 18.8%-59.4%), including 8.3% (95%CI: 1.0%-27.0%, n = 2) of patients achieving CR, 8.3% (95% CI: 1.0%-27.0%, n = 2) of patients achieving PR and 20.8% (95% CI: 7.1%-42.2%, n = 5) of patients achieving mCR/HI (Table 2). Median time-to- response (TTR) was 5 (95% CI: 2.00-NE) months.

All the 9 patients that were considered responders achieved responses within 4 cycles of AZA, and 7 (77.8%) of which were within 3 AZA cycles. 4 (44.4%) of the 9 responders were treated with AZA based on BSA. In patients who achieved a response, the median DOR was 12.0 months. 2 (8.3%) patients received subsequent haemopoietic stem cell transplantation (HSCT) treatment.

**Fig. 1:**
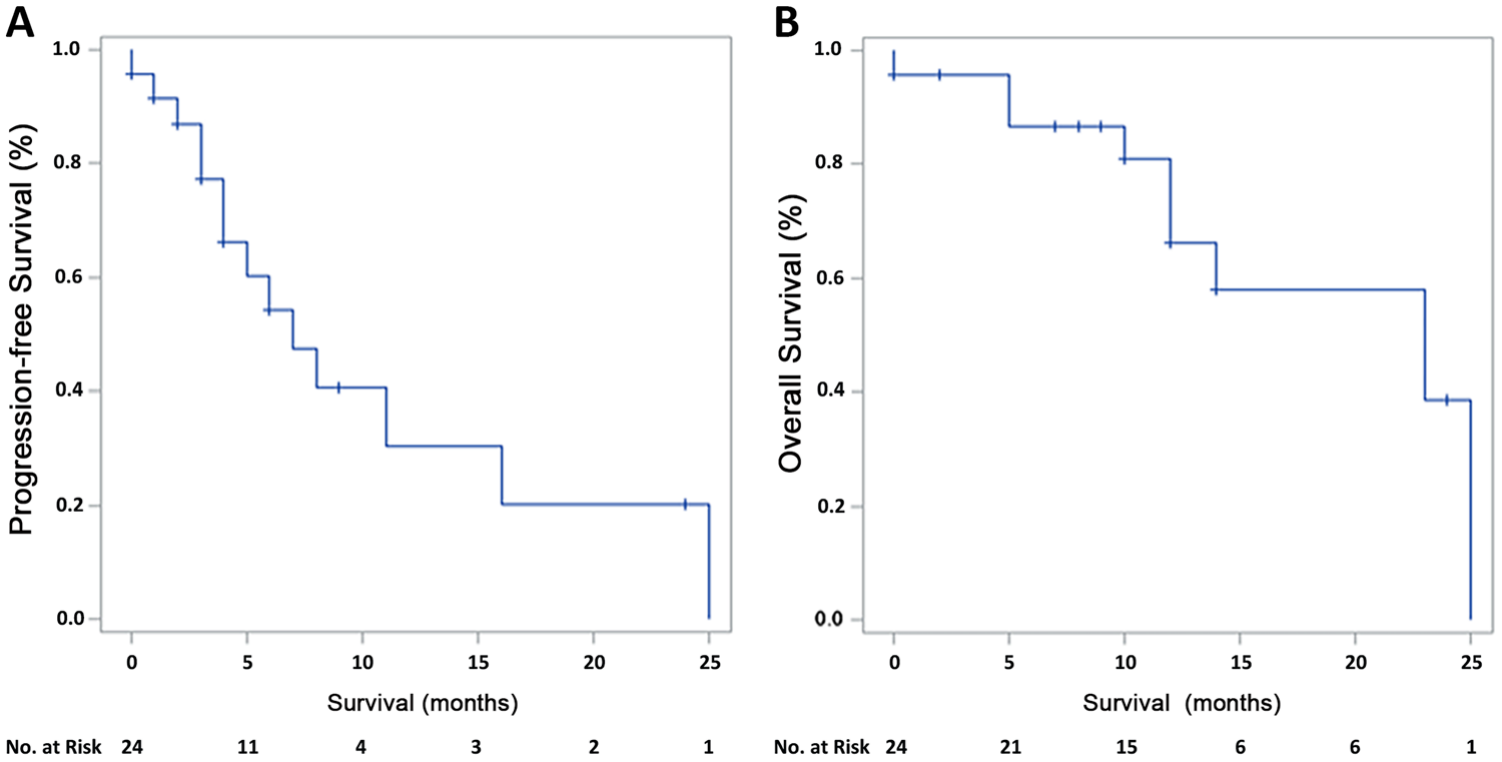
Progression free survival (**A**) and overall survival (**B**) of patients with CMML treated with azacitidine

After a median duration of follow-up of 14.0 months (range, 0.0–22.0 months), the median PFS was 7.0 (95%CI, 4.0–16.0) months, and the median OS was 23.0 (95% CI, 12.0-NE) months (Fig. 1). The 1-year PFS and 1-year OS rate were 30.5 (95% CI, 5.8%-55.1%) and 66.2% (95% CI, 43.1%-89.3%), respectively. The 1-year OS rate was 100% in the 5 patients who completed 6 cycles of AZA treatment.

**Fig. 2:**
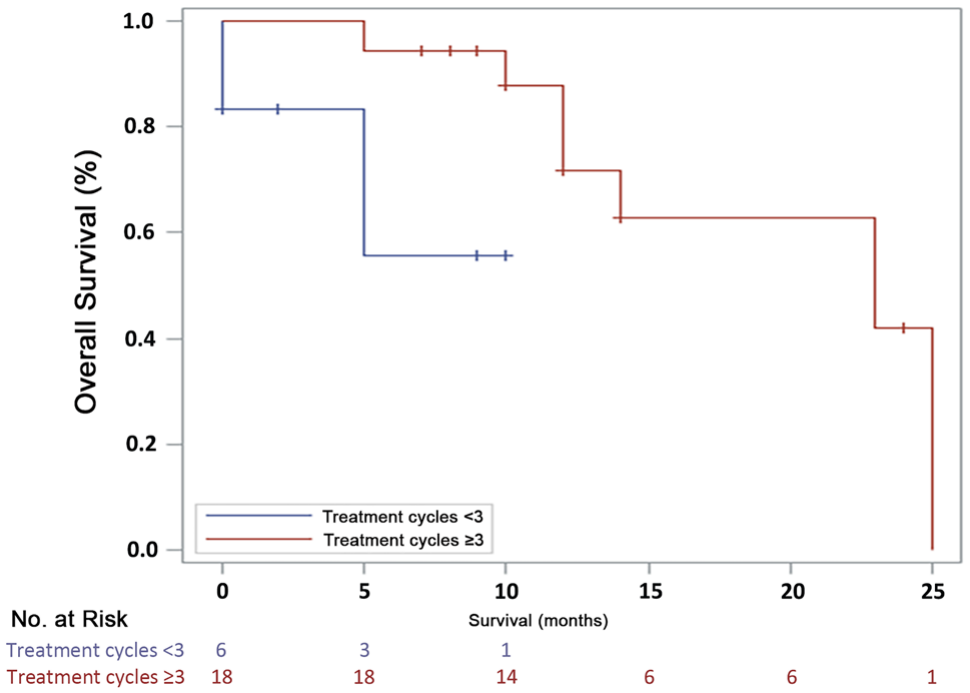
Overall survival by azacitidine treatment cycles (< 3 cycles vs ≥ 3 cycles)

We further analyzed the 1-year OS rate by stratifying for treatment cycles ≥ 3, aberrant karyotype, FAB or WHO 2016 subtype, peripheral hemoglobin < 80 g/L, and platelets < 100 × 10^9^/L. Univariate analysis revealed that ≥ 3 cycles of treatment was significantly associated with a longer overall survival compared with < 3 cycles of AZA treatment (*P* = 0.044, Fig. 2). No statistically significant association was observed in overall survival with other factors, such as objective response, aberrant karyotype, FAB or WHO 2016 classification, peripheral hemoglobin < 80 g/L, and platelets < 100 × 10^9^/L.

### Safety

AZA was generally well tolerated in all patients completing more than one cycle of treatment. Adverse events (AEs) were observed in 11 (45.8%) of 24 patients, and ≥ grade 3 AEs were reported in 9 patients (37.5%). The most common (> 10%) adverse events were thrombocytopenia (n = 7, 29.2%), pneumonitis (n = 4, 16.7%) and fever (n = 3, 12.5%). Serious adverse events were reported in 9 patients (37.5%); no fatal AE occurred. The majority of AEs were resolved or improved after management. The adverse events are depicted in Table 3.

**Table 3:** Adverse events

	**Any grade (≥ 5%), n (%)**	** ≥ grade 3, n (%)**
**Thrombocytopenia**	7 (29.2)	7 (29.2)
**Pneumonitis**	4 (16.7)	1 (4.2)
**Fever**	3 (12.5)	1 (4.2)
**Anemia**	2 (8.3)	2 (8.3)
**Pleural effusion**	2 (8.3)	0 (0.0)

## Discussion

To our knowledge, this is the first real-world report evaluating the efficacy and safety of AZA in CMML treatment based on a relatively large number of patients from multi-centers in China. In 24 CMML patients treated with AZA monotherapy or combination therapy, the ORR was 37.5% (8.3% CR, 8.3% PR, 20.8% mCR/HI), and the median OS was 23 months (95%, 12.0-NE). AZA was given for a median of 3 cycles (range, 1–8), and overall survival of patients with ≥ 3 cycles of AZA treatment was statistically significantly longer than patients with within 3 cycles (*P* = 0.044), under the situation where no statistical methods were used to adjust the multiplicity. Given the retrospective nature of this study, it is possible that this finding does not preclude that other patient characteristics may contribute to the improved OS among these patients. No significant association was observed in overall survival with other factors, such as objective response, aberrant karyotype, FAB or WHO classification, peripheral hemoglobin < 80 g/L, and platelets < 100 × 10^9^/L.

Initial reports of the efficacy of AZA in the treatment of CMML came from randomized trial studies on MDS patients with a limited number of CMML patients and the efficacy not being reported [22, 23]. In previous non-randomized clinical trials and retrospective observational studies, the overall response rate (ORR) of AZA in the treatment of CMML ranges from 20 to 60% [24–29]. Our data showed a comparable activity of an ORR of 37.5% (95% CI, 18.9%-59.4%) with previous reports in CMML patients. All the responders achieved remission within the first 4 cycles of AZA treatment, which is consistent with that 75% of responses occur within 4 cycles and 90% of responses occur within 6 cycles in previous MDS/MPN studies [32]. The overall survival for our cohort (median, 23 months) was similar to previous studies which report a range of median survival from 12 to 29 months [14–19].

Although a minimum of 6 cycles of AZA treatment is recommended [33], we observed a short AZA treatment with a median of 3 cycles in real-world practice in participating centers in this study. Similar small number of cycles of HMAs therapy has also been reported by Ma et al. with a median of four cycles in their cohort in Chinese patients [30]. The reasons may be complicated and related to the complex medical environment in China. One possible reason may be patients’ transferring to the subordinate hospitals or local hospitals for treatment given that subcutaneous AZA could be administered in the outpatient department. This leads to the failure to obtain treatment records in participating centers and renders the low number of AZA treatment cycles.

Fixed dose of AZA dose schedule, also known as “flat” dosage, has been reported in CMML and MDS treatment [34, 35]. Pleyer et al. revealed that “flat dosage” was administered in 18% out of 458 AZA cycles in 48 CMML patients, with 17% of the dose reduction related to an adverse event [35]. However, we observed that the fixed dose of AZA treatment was commonly applied with relative longer treatment courses in clinical practice in China, mostly without recordings of adverse events related to dose reduction. This phenomenon may be partially attributed to economic reasons considering that drug waste could be caused by AZA dosing based on BSA. Given the limited sample size in this study, further study is needed to investigate the effect of dose reduction on the efficacy and survival in Chinese patients with CMML.

The prognosis of CMML patients varies. Although attempts have been made to identify factors predicting survival in CMML, the prognostic value of factors remains inconsistent and controversial. Adès et al. reported that peripheral WBC > 13 × 10^9^/L was associated with worse OS in CMML patients treated with AZA [36].In a large cohort of retrospective multicenter study including 280 CMML patients from Latin American, González et al. revealed that Hb ≥ 8- < 10 g/dL, Hb < 8 g/dL, poor karyotypes, WHO 2016-CMML-1, and CMML-2 were independent adverse clinical factors associated with poor survival of CMML [11]. In the present study, clinical and laboratory factors, such as aberrant karyotype, FAB or WHO classification, peripheral hemoglobin < 80 g/L, and platelets < 100 × 10^9^ /L were not correlated to the survival of patients, which may partially relate to the limited sample size in this study.

In general, AZA was well tolerated with acceptable toxicity in most CMML patients, with commonly observed adverse events including thrombocytopenia, pneumonitis, bone marrow hypocellular and fever. Toxicities were similar to those observed in other studies using AZA in CMML treatment [35, 37].

Our study carries drawbacks given its retrospective nature with selection bias inevitable. Given the low incidence of CMML, only 24 patients fulfilled the inclusion criteria in five participating centers. The limited sample size and heterogeneity of patients’ characteristics and treatment could have an impact on the efficacy and survival, so the results should be interpretated with caution However, this study still retains the advantage of providing real-world data on the use of AZA in patients with CMML in China, for the first time in a relatively large Chinese patient cohort.

## Conclusions

In conclusion, our observations provided valuable real-life data in China on the treatment schedules, efficacy and safety of AZA in the treatment of CMML.

## Data Availability

The datasets generated during and/or analysed during the current study are available from the corresponding author on reasonable request.
